# Group Independent Component Analysis and Functional MRI Examination of Changes in Language Areas Associated with Brain Tumors at Different Locations

**DOI:** 10.1371/journal.pone.0059657

**Published:** 2013-03-26

**Authors:** Liya Wang, Dandan Chen, Xiaofeng Yang, Jeffrey J. Olson, Kaundinya Gopinath, Tianning Fan, Hui Mao

**Affiliations:** 1 Department of Radiology and Imaging Sciences, Emory University School of Medicine, Atlanta, Georgia, United States of America; 2 Center for Systems Imaging, Emory University School of Medicine, Atlanta, Georgia, United States of America; 3 Department of Radiology, Baoan Hospital, Shenzhen, Guangdong, China; 4 Department of Physics, Emory University, Atlanta, Georgia, United States of America; 5 Department of Neurosurgery, Emory University School of Medicine, Georgia, United States of America; University of Maryland, College Park, United States of America

## Abstract

**Object:**

This study investigates the effect of tumor location on alterations of language network by brain tumors at different locations using blood oxygenation level dependent (BOLD) fMRI and group independent component analysis (ICA).

**Subjects and Methods:**

BOLD fMRI data were obtained from 43 right handed brain tumor patients. Presurgical mapping of language areas was performed on all 43 patients with a picture naming task. All data were retrospectively analyzed using group ICA. Patents were divided into three groups based on tumor locations, i.e., left frontal region, left temporal region or right hemisphere. Laterality index (LI) was used to assess language lateralization in each group.

**Results:**

The results from BOLD fMRI and ICA revealed the different language activation patterns in patients with brain tumors located in different brain regions. Language areas, such as Broca’s and Wernicke’s areas, were intact in patients with tumors in the right hemisphere. Significant functional changes were observed in patients with tumor in the left frontal and temporal areas. More specifically, the tumors in the left frontal region affect both Broca’s and Wernicke’s areas, while tumors in the left temporal lobe affect mainly Wernicke’s area. The compensated activation increase was observed in the right frontal areas in patients with left hemisphere tumors.

**Conclusion:**

Group ICA provides a model free alternative approach for mapping functional networks in brain tumor patients. Altered language activation by different tumor locations suggested reorganization of language functions in brain tumor patients and may help better understanding of the language plasticity.

## Introduction

Blood oxygenation level dependent (BOLD) functional magnetic resonance imaging (fMRI) is widely used in presurgical planning for brain tumor resection [1], [2] to non-invasively determine functional brain regions so that operable margins can be defined. One of the major applications of fMRI in presurgical planning is to identify areas relevant to speaking, reading and other language activities. In addition to assessing the potential risk of functional impairment associated with surgery, the important question for presurgical mapping of language areas of brain tumor patients is whether language areas are displaced due to the presence of a tumor that presses normal tissues, especially large tumors invading adjacent areas. This can be challenging as the substantial reorganization of functional areas may also take place during the growth of tumor. Understanding the functional reorganization is not only important for mapping the resection margin, but also informative for predicting functional outcomes of the surgery and preparing a strategy for rehabilitation. Furthermore, mapping of the eloquent brain structures of tumor patients, e.g. language network, may also provide critical insights on the mechanism of functional reorganization and brain plasticity.

Most current clinical practices of presurgical brain mapping with fMRI uses a general linear model (GLM) to carry out the voxel-wised statistical analysis of BOLD signal time courses and hemodynamics response associated with brain activation [3], [4]. However, there is increasing evidence that abnormal blood supplies in brain tumor can alter the hemodynamic responses that may deviate from the empirical models derived from healthy controls [5]–[8]. On the other hand, independent component analysis (ICA) is a data driven and pattern recognition, or model-free, approach that is capable of determining high probability components of signal time course patterns without the hypothesis-driven reference functions or predefined seeds required in the model-based approaches [9], [10]. ICA has gained increasingly interests in fMRI data analysis as applied in many early studies, but most of them focused on either normal control groups or an individual patient [11], [12]. Since interrogation of fMRI signal time courses with ICA is independent of the designed stimulation paradigm or pre-assumption of an activation pattern, it minimizes the mode-fitting bias when extracting the activation patterns that may be altered from the normal or predicted forms. Therefore, it is more appropriate for analyzing functional reorganization associated with the diseases, such as brain tumors, which may cause alteration of BOLD signal time courses. However, there are very few studies that have investigated the utility of ICA in mapping functional areas of brain tumor patients and its potential in clinical applications in presurgical mapping of language areas, especially with a group analysis approach.

The current study is to examine the functional reorganization of language areas in brain tumor patients with different tumor locations using group ICA. We investigated the effect of the tumor location on changes of language functions and the utility of group ICA by comparing observed alterations in language network in our presurgical fMRI mapping of these tumor patients as well as previous reports in the literatures[11], [13]–[16]. Our re-examination of changes in language functions in different locations of brain tumor patients with this model free approach thus may lead to better understanding of the lesion induced language plasticity, which is important to the reliable mapping of the language areas in brain tumor patients.

## Materials and Methods

### Brain Tumor Patients

This study was approved by Emory Institutional Review Board for the human subject research. Written informed consents were obtained from all participants before the study. Forty three patients (20 females; age range = 21–63 years; mean = 45.3) who were diagnosed with primary brain tumors and underwent presurgical fMRI mapping of language areas were included in this study. They were all right-handed. fMRI data were analyzed retrospectively. Tumor locations were determined based on T_2_ weighted fast spin echo imaging and the contrast enhanced T_1_ weighted spin echo imaging. Pathology results were obtained after the tumor removal. Tumors were graded according to the World Health Organization (WHO) brain tumor grading system (2007) (a four-tiered histological grading guideline that assigns a grade from I to IV with the Grade I being the least aggressive and IV being the most aggressive) [17], [18]. All patients in our study did not have any other neurological diseases. To investigate the effect of the tumor location on functional changes of the cortical presentation of language functions, we divided patients into three groups: 1) the group with tumors on the right hemisphere (n = 12), including frontal (n = 5), temporal (n = 4) and parietal lobes (n = 3) as RH Group; 2) the group with tumors in the left temporal lobe (n = 16) as LT Group; and 3) the group with tumors in the left frontal lobe (n = 15) as LF Group. Left and right hemispheres are delineated by the median plane (median longitudinal fissure). Tumor boundaries separating frontal, temporal and parietal lobes were determined based on anatomic topography, i.e. the central sulcus separates frontal lobe from parietal lobe; the lateral sulcus separates frontal lobe from temporal lobe on the lateral surface of human brain; the lateral sulcus (sylvian fissure) is the most lateral boundary separating the parietal lobe from the temporal lobe and the parieto-occipital sulcus separates the parietal lobe from the occipital lobe. If a tumor occupies over one lobe, its location was defined according to the location of the larger proportion.

### MRI Data Acquisition

All MRI data were recorded on a 3T MRI scanner (Siemens MagnetoTim/Trio, Siemens Healthcare) using a standard phased array head coil. A routine clinical brain MRI protocol, including T_1_ and T_2_ weighted spin echo and gradient echo sequences as well as gadolinium contrast enhanced T_1_ weighted spin echo imaging, was performed on each patient for tumor localization and characterization.

For mapping language cortices, patients were instructed to perform a picture naming task overtly. This task requires a subject to generate a sentence, or a word if time is limited, to name the presented animals drawn in black and white lines [19]. A block design paradigm with a time course of 90 points (4 ON and 5 OFF blocks) was used with self-paced counting as the baseline or OFF condition. The timing, fixation and picture presentation, as well as task ON or OFF block, were given to the patient using visual display delivered from a stimuli control program via. an LCD screen mounted on the head coil. BOLD fMRI data with a time series of 90 volumes were recorded using a single-shot T_2_* weighted gradient echo EPI sequence. Image acquisition parameters included: TR/TE of 3000/35 ms, Field of View (FOV) of 240 mm, matrix of 64×64 and 25 slices with slice thickness of 5 mm without gap. In addition, high resolution T_1_ weighted multiplanar gradient recall (MPREG) and T_2_ weighted fast spin echo MRI scans were collected to obtain anatomic images for co-registration of functional maps.

### Image Procession and Data Analysis

fMRI data were processed and analyzed using the program of Multivariate Exploratory Linear Optimized Decomposition into Independent Components (MELODIC) implemented in the software of FSL (FMRIB, Oxford University, http://www.fmrib.ox.ac.uk/fsl/). Before applying the ICA, all functional images were preprocessed using following procedures: motion correction to align BOLD images in the time series; skull striping to remove non-brain tissue; spatial smoothing of images using a Gaussian kernel of full-width- at-half-maximum (FWHM) of 6 mm. Gaussian-weighted high pass temporal filtering was applied for noise reduction [20].

Tensor-ICA was applied to perform group analysis of fMRI data from three defined groups. In the tensor-ICA approach, BOLD image data for the whole group was decomposed into a set of independent components that characterize the spatial, temporal, and subject domains. Tensor-ICA assumes that the temporal response pattern is the same across the population and provides a single decomposition for all original data sets. Based on the variance of the residuals, the highly non-Gaussian components were derived. This model-free method is able to extract activation maps from individual components or patterns, time courses, and estimates of the signal variation across the population [21]. To identify voxels with statistical significant contribution, activation maps of derived independent component were scaled to the spatial *z*-scores of standard deviations of the map [22], [23]. A threshold of *Z* >2.0 was used for visualization of activation maps and volumetric comparison of maps of each independent component [24].

The functional maps obtained from ICA were evaluated to examine the language areas involved in the picture naming task. Relevant independent components and corresponding activation maps were determined based on three criteria: 1) regions of activation that are similar to the language activation patterns reported previously [25]–[27]; 2) the signal time course that closely matches the task paradigm; 3) the probability rank of the component that gives statistical significance. The primary activation components selected from each group, which were the best fitted component from all estimated ICA spatial maps, were co-registered to the standard brain images in the Talairach brain atlas [28]. Voxels with above selected thresholds (*Z* >2.0) then were displayed to demonstrate the regions associated to overt naming tasks.

Quantitative analysis of the activation area was carried out in different groups using the defined volumes of interest (VOIs). In briefly, a total of 6 VOIs known to associate with a picture naming task used in this study [25]–[27], [29] were extracted from the primary activated map of each group based on the Brodmann areas (BAs). They include inferior frontal gyrus in the extended Broca’s area (BAs 44, 45, 47, 9 and 6) on the left hemisphere (VOI 1), inferior frontal gyrus (BA 47) in the contralateral Broca’s area (VOI 3), superior temporal gyrus (BAs 21 and 22) in left Wernicke’s area (VOI 2), anterior cingulate and medial frontal gyrus (BAs 32, 8 and 6) on the left hemisphere (VOI 4), superior parietal lobule (BA 7) on the left hemisphere (VOI 5) and the right hemisphere (VOI 6), respectively. Six VOIs were defined on different slices of the MRI template by experienced neuroradiologists and superimposed on activation maps of each group, resulting in a total of 18 VOIs for three groups studied. In order to quantify the activated size of VOIs using an automatic procedure, the mean image of the activation map from each group was realigned to an anatomically standardized stereotactic template using a validated registration algorithm with a nine-parameter rigid body transformation [30]. The number of voxels in each VOI for each group was counted to compare the changes in different VOIs between groups.

For comparison with group ICA, single subject and single-session ICA was applied to each individual patient to derive group comparison. This was done with data from individual activation maps selected from the most appropriate task related IC components of the single subject ICA based on the same criteria as described above in tensor ICA. Selected individual action maps were then submitted to AFNI for statistical group comparisons using one-way ANOVA that yielded subject-specific voxel number counts for each group. The number of voxels classified as active (*Z* >2.0) within each of the VOIs for each subject was tabulated. The numbers of overlapping voxels within each VOI between different groups from the independent components were used to indicate the degree of correlation in activation patterns between each two groups.

Laterality index (LI) was used to assess language lateralization in the extended Broca’s area only, i.e. VOI 1 and VOI 3 in each group. The LI was calculated using the following equation [31]:
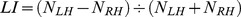
(1)in which *N_LH_* and *N_RH_* are the numbers of overlapping voxels in Broca’s area, i.e. frontal area on left and right hemispheres respectively. Positive LI values indicate left dominant language function and negative values indicate right dominant language function.

All statistical analyses were carried out using the software Statistical Package for the Social Sciences (SPSS) version 19.0 (SPSS Inc. Chicago, IL, USA) [32]. The age, gender, tumor grade and tumor volume of patients in three groups were compared using one way ANOVA analysis. The results with *P*<0.*05* were considered statistically significant.

## Results

### Demographic and Clinical Characterization of Brain Tumor Patients

The demographic and clinical data from patients included in this study were summarized in **Table 1** **.** There was no statistical significant difference in age, gender, tumor grade and volume between three groups based on the one-way ANOVA analysis. Typically, low grade tumors were non-enhanced lesions in post-contrast T_1_ weighted MRI with averaged tumor size as small as 7 cm^3^. High grade tumors presented as an enhanced mass and considerable edema around the tumor with averaged tumor size as large as 52 cm^3^. LT group, i.e., tumors in the left temporal area, consisted of 16 patients, including three low grade (Grade I or II) and thirteen high grade (Grade III or IV), with tumors involving insular, parts of temporal and middle frontal gyrus, as well as precentral and postcentral gyrus on the left hemisphere. LF group, i.e., tumors in the left frontal area, consisted of 15 patients, including three low grade and twelve high grade tumors. Tumors in LF group were found in parts of frontal gyrus, inferior parietal lobule, cingulated, precentral and postcentral gyrus on the left hemisphere. The group with tumors on the right hemisphere (i.e., RH group), defined as a control group, included five low grade and seven high grade tumors. Locations of these tumors varied within this group as they were found in different parts of the right frontal lobe, including precertral gyrus (n = 5), insular gyrus (n = 4), postcentral gyrus and anterior cingulate gyrus (n = 3).

**Table 1:** Demographic and Clinical Information about Patients.

	Left Frontal Tumor	Left Temporal Tumor	Right Hemisphere Tumor
**Case Number**	15	16	12
**Grade** **(High/Low)**	12/3	13/3	7/5
**Age**	50.2	48.3	38.5
**Gender (F/M)**	5/10	6/10	9/3
**Hemisphere**	Left	Left	Right
**Tumor Volume (cm^3^)**	54.6	48.6	46.4
**Brain Areas**	CGG, PRCG, POCG, IFG, MFG,SFG, IPL	ISG, STG, MTG, ITG, MFG, IPL,PRCG, POCG	SFG, MFG, IFG, ACGG, ISG, MTG, STG, PRCG, POCG, CGG
**Tumor Associated** **Brodmann Area**	2, 3, 4, 6, 8, 9, 24, 31, 32, 40 (L)	13, 21, 22, 19, 6, 20, 40 (L)	8, 9, 24, 32, 47, 13, 21, 28,37, 38, 43 (R)
**Center of Mass from All** **Tumors in MNI (mm)**	MFG (−30, 4, 38)	MTG (−56, −22, −14)	ISG (32, −10, 12)

Notes: CGG: cingulate gyrus; PRCG: precentral gyrus; POCG: postcentral gyrus; IFG: inferior frontal gyrus; MFG: middle frontal gyrus; SFG: superior frontal gyrus; IPL: inferior parietal lobule; ISG: insular gyrus; STG: superior temporal gyrus; MTG: middle temporal gyrus; ITG: inferior temporal gyrus; ACGG: anterior cingulate gyrus.

### Activation Maps of Different Tumor Groups Obtained from ICA

**Figure 1:**
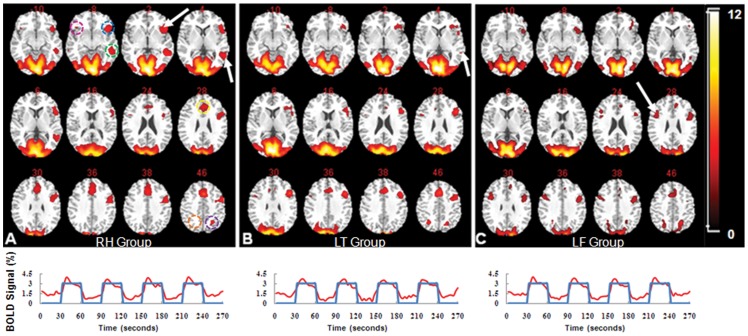
Language areas of three different groups of brain tumor patients identified by group ICA. Activation maps in three different groups from selected slices demonstrate the cortical structures activated by the overt picture naming task in brain tumor patients with different location. **A**: RH Group with right hemisphere tumor, which shows the putative language areas, including the Broca’s and Wernicke’s areas in the left hemisphere (indicated by arrows). **B**: LT Group with tumors located in the left temporal areas. Significant decreases of activations in the Wernicke’s area were seen (arrow indicated) in this group. **C**: LF Group with tumors in the left frontal areas, noticing compensatory language area shifted to the upper of Broca’s area in the right hemisphere (arrow). Activation maps were obtained with a threshold of z >2.0. All images are displayed in radiological convention. Time courses of independent components corresponding to the activation maps in **A–C** are shown under the activation maps. ICA was able to identify language activation in all three groups. Cortical structures and regions selected for comparison of activation patterns of different groups and measurement of the volumes of interest (VOIs) marked with different colors, Blue: VOI 1; Green: VOI 2; Magenta: VOI 3; Yellow: VOI 4; Purple: VOI 5; Orange: VOI 6.

Functional maps of language activation evoked by the picture naming task were obtained successfully from all three groups of patients using grouped ICA. Among more than 20 independent components in each group derived from the ICA, we selected three independent components (one for each group) that not only had a signal time course closely matching the task paradigm of 4 On and 5 Off blocks but also had the highest probabilities. **Figure 1A, B and C** show the language specific activation maps of three groups, respectively. The presented time courses obtained from each group were averaged from all activated voxels that had the similar pattern, although it is expected that regional differences in the response time and BOLD signal intensity may vary in individual patients. The detailed anatomical assignments of activated cortical structures in each group are listed in **Table 2**.

**Table 2:** Locations of Activated Language Areas Identified by fMRI.

Volumes of Interest	Area	BAs	RH Group	LT Group	LF Group
VOI 1	IFG	47	(−36, 32, −10)	(−36, 32, −10)	(−36, 32, −10)
	IFG	45	(−42, 20, 6)	(−42, 20, 6)	–
	IFG	44	(−52, 16, 14)	(−52, 16, 14)	–
	IFG	9	(−46, 16, 28)	(−46, 16, 28)	(−46, 16, 28)
	MFG	6	(−42, 6, 40)	(−42, 6, 40)	(−42, 6, 40)
	Centre in MNI		IFG (−52, 20, 14)	MFG (−48, 16, 24)	IFG (−46, 4, 26)
VOI 2	MTG	21	(−56, −28, −2)	–	–
	STG	22	(−56, −46, 6)	–	–
	Centre in MNI		MTG (−60, −42, 0)	–	–
VOI 3	IFG	47	(40, 22, −8)	–	–
	IFG, MFG	9	–	(40, 10, 28)	(40, 10, 28)
	MFG(PRCG)	6	–	–	(50, 6, 38)
	Centre in MNI		IFG (40, 22, −8)	IFG (40, 10, 28)	PRCG (44, 8, 34)
VOI 4	ACGG, CGG	32	(−2, 38, 20) (−2, 26, 38)	(−2, 26, 38)	(−2, 18, 38)
	MFG, SFG	8	(−2, 38, 38) (0, 20, 50)	(0, 26, 42)	(−2, 24, 42)
	SFG	6	(−2, 12, 56)	(−2, 14, 54)	(4, 12, 56)
	Centre in MNI		MFG (0, 22, 44)	SFG (0, 18, 48)	SFG (0, 16, 50)
VOI 5	SPL(PCG)	7	(−22, −60, 50)	(−20, −62, 52)	(−20, −62, 50)
	Centre in MNI		(−24, −62, 50)	SPL (−22, −62, 50)	PCG (−20, −62, 50)
VOI 6	SPL(PCG)	7	(24, −60, 50)	(26, −60, 52)	(22, −62, 48)
	Centre in MNI		PCG (24, −60, 50)	SPL (28, −60, 48)	PCG (22, −62, 48)

Notes: BAs: brodmann areas; RH Group: group with right hemisphere tumors; LT Group: group with left temporal tumors; LF Group: group with left frontal tumors; VOI 1–6: different volumes of interest; IFG: inferior frontal gyrus; PRCG: precentral gyrus; MFG: middle frontal gyrus; MTG: middle temporal gyrus; STG: superior temporal gyrus; ACGG: anterior cingulate gyrus; CGG: cingulate gyrus; SFG: superior frontal gyrus; SPL: superior parietal lobule; PCG: precuneus gyrus.

For this study, the activation map of the group with right hemisphere tumors, not healthy controls, was used as a putative language map for evaluation of tumor induced changes in language areas. It suggests that tumors on the right hemisphere have minimal effect on the cortical organization as the language activation map of this group exhibited a similar activation pattern of the healthy controls as reported in the early studies [33], [34]. Consistent with those previous reports, we observed the activated language areas located mainly in the left Broca’s area (Brodmann areas 44, 45, and 47) and in the left Wernicke’s area (BAs 22 and 21). Small activated areas were observed in the inferior frontal gyrus (BA 47) of the right hemisphere. In addition, activation was observed in the areas of BAs 32, 8, and 6, including anterior cingulated, superior and middle frontal gyrus, and BA7 (superior parietal gyrus) on both hemispheres. **Figure 1** presents activated language areas in different groups. Although activations in the occipital visual cortices appear to be similar in different groups, the putative language areas were changed in LT and LF Groups (tumors in the left hemisphere) in comparison to those of RH Group (tumors in the right hemisphere).

### Changes of Activation Areas Associated with Left Hemisphere Tumors

**Figure 2:**
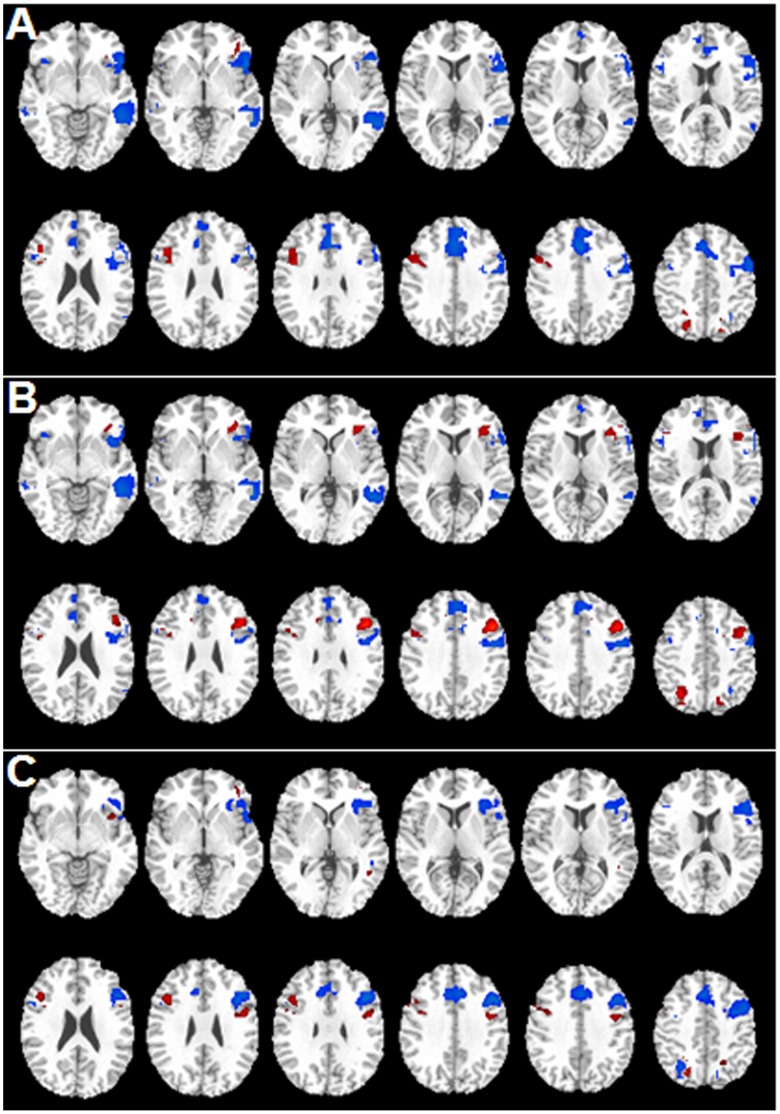
Tumor location associated changes in language areas. Differences in activation patterns between groups are illustrated in the selected slices using maps obtained from subtractions of activation maps of two different groups with positive values in red and negative values in blue. **A:** The different map obtained from the group with left frontal tumors subtracting the group with tumors in the right hemisphere; **B:** The different map obtained from the group with left temporal tumors subtracting the group with tumors in the right hemisphere; **C:** Subtraction map from the group with left frontal tumors subtracting the group with left temporal tumors.

Both Broca’s and Wernicke’s areas showed reduced activations in LT and LF Groups, while a significant increase of activations in the right Broca’s area, i.e. BA 47, mainly extending to the inferior frontal gyrus, was observed in LF Group. Furthermore, changes in functional areas were dependent on the location of tumors. The spatial differences in activation patterns between two groups are shown in **Figure 2** **,** which compared the different groups by directly subtracting their co-registered activation maps. In addition to observing that Broca’s area (BAs 44, 45, and 47) and Wernicke’s area (BAs 22 and 21) exhibited reduced activations in both LF and LT Groups (**Figure 2A and 2B**) compared to RH Group, left frontal locations of tumors had a more pronounced effect in the Broca’s area (**Figure 2C**). Increase of activations in the right frontal lobe, e.g. inferior frontal gyrus in BA 47 on the right hemisphere was found in both LF and LT Groups (**Figure 2A and 2B**) with a higher increase found in LF Group when compared to LT Group. Interestingly, activations in Broca’s area (BA 44, 45, and 47) were diminishing, but preserved in certain degree in group with left temporal tumors (LT Group). Increased activation was found in the areas anterior to nearby tumors in the group with left frontal tumors (LF Group). Observed difference in activation between LT and LF Groups showed stronger effect of left frontal tumors in the Broca’s area (**Figure 2C**). Increased activations were also observed in right frontal areas (BA 47) of LF Group when compared to LT Group as shown in **Figure 2C**.

**Figure 3:**
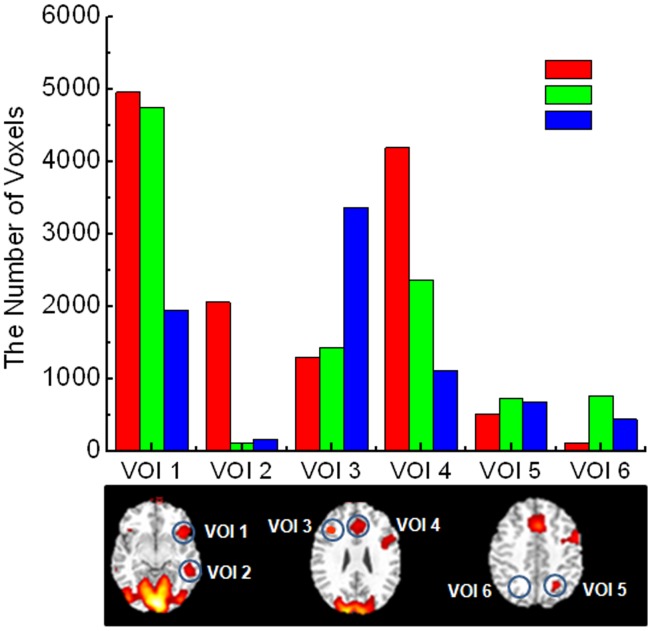
Variation of BOLD activation as the results of tumors in different locations. The level of language activation in three different groups of patients is demonstrated by the numbers of activated voxels in selected VOIs. Red bar indicated the number of voxels on VOIs in the group with tumors in the right hemisphere (RH Group); green bar indicated the number of voxels on VOIs in the group with tumors located in the left temporal area (LT Group); and blue bar indicated the number of voxels on VOIs of the patients with tumors in the left frontal area (LF Group). BOLD activation decreased progressively on VOI 1 and VOI 4 from RH Group, LT Group to LF Group. In contrast, activation increased progressively on VOI 3 in the right hemisphere. BOLD activation on Wernicke’s area (VOI 2) was mostly diminished in LT Group, but was increased in the superior parietal gyrus on both hemispheres (VOI 5 and VOI 6).

To quantitatively assess the regions involved in the picture naming task in different groups, activated voxel numbers in all six VOIs from each group were counted and compared in **Figure 3**. A total of 4965 activated voxels were measured in the extended Broca’s area (VOI 1) of RH Group. In comparison, the numbers of the activated voxels in the same VOI were 4750 in LT Group and only 1944 in LF Group. In the Wernicke’s area (VOI 2) of the left hemisphere of RH Group, a total of 2056 activated voxels were counted. However, activated voxels dropped to 114 in LT Group and 163 in LF Group, respectively. The anterior cingulate cortex in BAs 32, 8, and 6 (VOI 4), which is an important part of the language network, had a total of 4194 activated voxels in RH Group. In comparison, only 2368 activated voxels were counted in the anterior cingulate cortex of LT Group. The total number of activated voxels further decreased to 1116 in LF Group.

The tumor induced language reorganization was also evidenced by the observation of increased activation in several regions of the right hemisphere in patients with left hemisphere tumors. A total of 3366 activated voxels in the inferior and middle frontal gyrus (BAs 9 and 6, i.e. VOI 3) in LF Group and 1424 activated voxels in LT Group were observed, respectively, while only 1300 activated voxels were seen in the right inferior and middle frontal gyrus (VOI 3) in RH Group. Furthermore, in the precuneus gyrus (BA 7, VOI 5 and VOI 6) of the left and right hemispheres, 677 and 445 activated voxel numbers were measured from LF Group, while 728 and 768 activated voxels were measured in LT Group. In comparison, fewer activated voxels (517 and 112) were found in VOI 5 and VOI 6 in RH Group. The tumor induced language reorganization was also evidenced by observation of increased activation region on the right hemisphere of patients with left hemisphere tumors. A total of 116 activated overlapping voxels in inferior frontal gyrus (BA 47, i.e. VOI 3) in LF Group and 95 activated overlapping voxels in LT Group were observed, respectively, while only 90 activated voxels were seen in right inferior gyrus (VOI 3) in RH Group.

To compare and validate the results from group ICA, we also tested the approach of deriving group comparison results by analyzing each individual patient with single subject and single-session ICA. This was done with data from individual activation maps selected from one of the components of the single subject ICA. Statistical group comparisons using one-way ANOVA yielded subject-specific voxel number counts for each group. The numbers of overlapping voxels between different groups from the independent components were used to indicate the degree of correlation in activation patterns between each two groups. The results from individual ICA and one-way ANOVA analysis showed similar changes of activation patterns between RH Group and LF Group, RH Group and LT group, as observed in the results from group ICA analysis (**Figure 3** **)**. There were statistical differences between RH and LF Groups (*P*<0.001) in right Broca’s area (VOI 3) between RH and LT Groups (*P = *0.05), and between LT and LF Groups (*P = *0.05). Broca’s area **(**VOI 1) had different activation levels between two different groups, but the statistical difference showed only between RH and LF groups (*P = *0.05). In Wernicke’s area (VOI 2), statistical significant reduction of activation was observed in LT Group comparing to RH Group (*P = *0.05). The plot comparing these results can be found in Supporting Information **(Figure S1 in File S1)**. The degrees of differences of group comparisons are less pronounced in the data from individual ICA analysis, suggesting that group ICA analysis may be more sensitive in identifying the differences.

### The Lateralization of Language Areas in Patients with Brain Tumors

**Figure 4:**
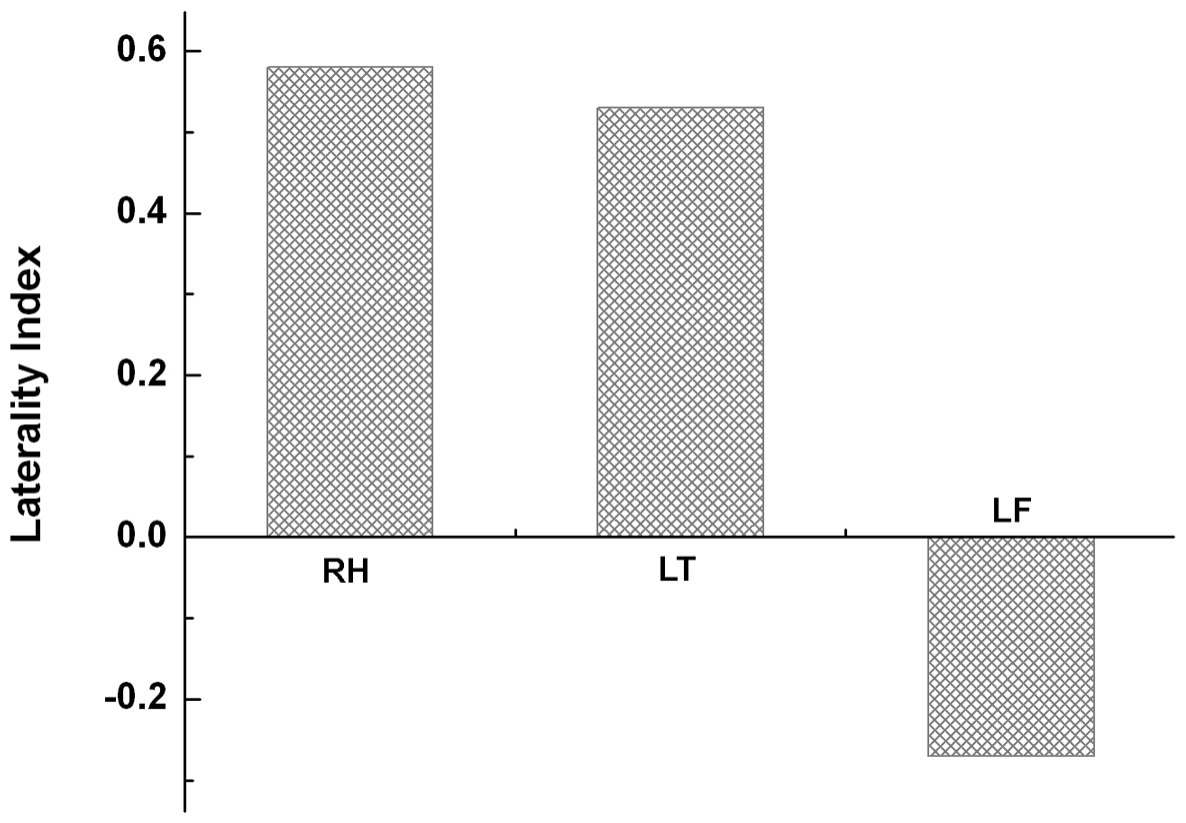
Tumor location associated changes of language lateralization. Left lateralization of language areas in patients with tumor located on the right hemisphere (RH Group) is indicated by predominant language activation in the left Broca’s area. Left lateralization was also preserved in patients with left temporal tumors (LT Group), however, it is less significant than that of RH group.). In contrast, right lateralization of language areas (sampled in Broca’s area) was observed in patients with eft frontal tumors (LF Group).

The reorganization of language areas is also described Laterality Index (LI), which is a quantitative comparison of hemispheric domination of activated areas. In the current study, LI was calculated based on the number of activated voxels in the extended Broca’s area on both hemispheres, i.e. VOI 1 and VOI 3. LI of patients with tumors in the right hemisphere is 0.58, which is higher than that of the group with left temporal tumors (LI = 0.53). Remarkably, patients with left frontal tumors exhibited a significant shift of language lateralization with a LI of −0.27, revealing that the language function is right hemisphere dominant in this group as indicated by a negative LI [13]. LIs of all three groups are compared in **Figure 4**.

## Discussion

Currently clinical applications of BOLD fMRI in patients, such as stroke and tumor, are typically using GLM analysis, mainly using readily available analysis tools, such as SPM, AFNI or BrainVoyager [13], [14], [16], [35]–[37]. It has been increasingly recognized that abnormal physiological conditions in patients may interfere the fMRI examinations in clinical practices. Physiological and biological changes caused by a tumor, such as neovascularization, mass effect and edema, can alter blood flow or induce a neurovascular uncoupling effect, and therefore change the hemodynamic responses from activations in functional areas [38], [39]. Earlier study has shown that the large tumor can disrupt blood flow in the language dominant hemisphere [38]. It is also found that BOLD effect is sensitive to the altered regional blood flow of high grade tumors [40]. When presurgical brain mapping for brain tumor patients uses relatively simple statistical analyses with empirical models of hemodynamic responses derived from healthy controls, tumor induced alterations in BOLD signal time courses may interferer the accurate calculation of functional maps. Although the corrections and adjustments may be made in models, it cannot be done without better understanding of the mechanisms and patient specific information on the BOLD signal alterations.

As a model free approach, ICA presents a possible solution for this problem. Our study of analyzing changes of language areas of patients with brain tumor at different locations demonstrated that ICA provides a better and model-free probabilistic approach for analyzing fMRI data containing physiological interferences that disrupt model fitting in conventional model-fitting analysis methods. A couple of case studies of applying ICA approach in mapping functional areas in brain tumor patient has been reported earlier [11], [33]. It was found that ICA identified more activated voxels in putative language areas comparing the results obtained from GLM methods. Signals from sources not related to brain activations were isolated into different components. Our results provide additional evidence to support the notion from other studies [37], [41], [42] that ICA can be used to identify patterns of activation at the presences of image artifacts and physiological interferences related to the abnormalities. To our knowledge, we are the first time to analyze fMRI data of tumor groups with different location using group ICA. Since we only intended to assess whether the group ICA analysis is feasible to derive observations in patients with individual differences in tumor pathology, intra-session and individual variability in patients were not assessed.

Language is a complex system that typically involves two primary language components Broca’s area [43] and Wernicke’s area [44] and dynamic interplay between these two areas [45]. Observed changes of language activation patterns in patients with brain tumors in different cortical locations provide a strong evidence of lesion induced interruptions and reorganization of the language network. Furthermore, our results demonstrated the association between tumor locations and their effect on specific cortical structures within the language network of Broca’s and Wernicke’s areas.

In this study, the control group is patients with brain tumor on the right hemisphere instead of healthy individuals with assumption that 97% of right-handed people have their "natural" tendency of language processes on the left hemisphere. Therefore, Broca area and Wernicke area are affected mainly by intracranial masses on the left hemisphere in the strictly right-handed patients. This is different from other previous studies which chose normal healthy volunteers as controls [46]. The rationale is that other brain activities related to mental and psychological process are similar in brain tumor patients. In our study, Broca’s area and Wernicke’s area are intact in the patients with tumors on the right hemisphere. However, BOLD fMRI measured activations were reduced in both areas in the patients with left hemisphere tumors as observed in the current study. Since the patients included in this study had language functions relatively intact as assessed in the pre-MRI exams, this observation suggests language functions originating in the tumor affected areas may be shifted to the regions nearby [47] or replaced by contralateral compensatory structures [25]. Indeed, substantial increase of right frontal activation (BA 9) was observed in the current study in patients with left frontal lesions. Changes in the lateralization of language areas also were supported by the negative LI value.

Besides functional changes in Broca’s and Wernicke’s areas, other areas involved in the language activities, for example BAs 9, 7, 6, 8, and 32, were also found altered in the current study. Activations associated with tumor induced functional reorganization in the contralateral upper Broca’s area have been considered to reflect a compensatory effect as the hemisphere with a tumor becomes compromised [38]. Since the growth of a low grade tumor and subsequent progression may take 2–5 years or longer, brain tumors in the left hemisphere can cause slow destruction of Broca’s area, while leaving speech relatively intact as other brain regions develop or “rewire” to compensate for the loss of language functions in primary language areas, such as Broca’s and Wernicke’s areas [36], [42]. Our results also exhibited similar reorganization based on the observed language activation pattern. Such functional plasticity exhibited in brain tumor patients is potentially important for predicting the functional outcome of tumor resections, and furthermore, for defining a possible rehabilitation strategy after surgery.

The current study has several limitations. For example, there is lack of behavioral tests. The sample size of each group was limited as only a small numbers of patients with right and left frontal tumor were available from the existing patient populations. In addition, we did not evaluate changes on language network based on the effect of the duration of tumor presence due to weak statistical powers at small sample sizes. Language lateralization of each group was assessed only using Laterality index of the extended Broca’s area. It should be noticed that control subjects with right hemisphere tumors in this study are mainly female while the other groups are mostly male. However, the comparison of gender difference using ANOVA showed the difference between groups, but it did not reach a statistical significance (P = 0.065). Gender difference in language functions may contribute to different activation patterns. It is unclear whether there is a gender differences in functional compensation of brain tumor patients and beyond the scope and ability of the current study. These limitations can be considered in future investigations to better understanding the reorganization process of language functions and clinical implications of fMRI language mapping with ICA methods.

### Conclusion

BOLD fMRI and group ICA enabled mapping the language areas of the patients with brain tumors in different locations. Significant reorganization of language areas was present in patients with tumor in the left frontal and temporal areas, evidenced by reduction of the left hemisphere lateralization. More specifically, tumors in the left frontal region affect both Broca’s and Wernicke’s areas, while tumors in the left temporal lobe affect mainly Wernicke’s area. Information on language reorganization derived from fMRI and ICA can help better understanding the lesion induced language plasticity that is important to the reliable mapping of language areas of brain tumor patients. As a model-free approach, ICA is particularly useful in presurgical fMRI mapping of tumor patients with altered language networks.

## Supporting Information

File S1 (DOC)
