# Supplementary material for: Group Independent Component Analysis and Functional MRI Examination of Changes in Language Areas Associated with Brain Tumors at Different Locations
Source: PLoS One. 2013 Mar 26;8(3):e59657. doi: 10.1371/journal.pone.0059657 (PMC3608667; doi:10.1371/journal.pone.0059657)
Supplement: File S1 — (DOC) [file pone.0059657.s001.doc]

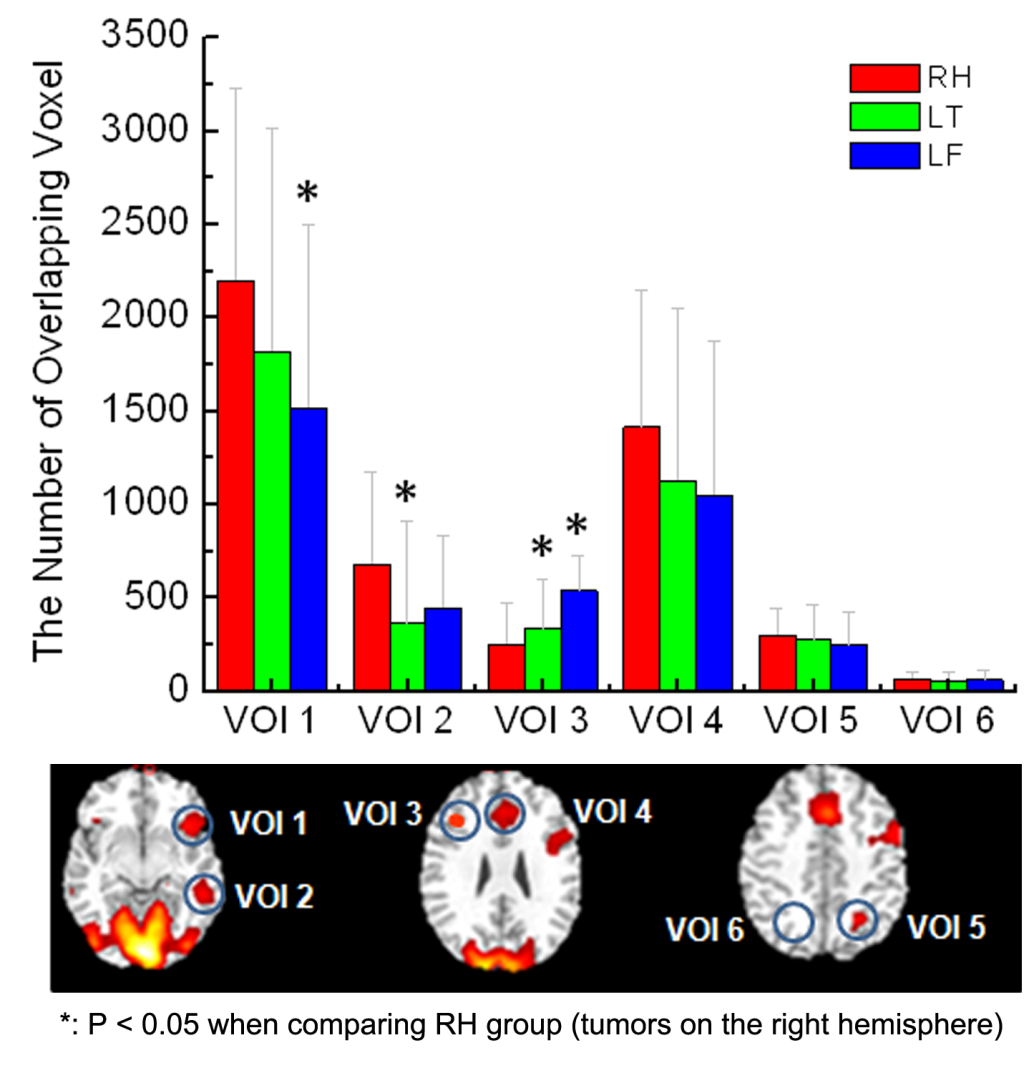


**Figure S1. Group comparison analysis was performed using ANOVA**

The level of language activation in three different groups of patients is demonstrated by the numbers of activated voxels in selected VOIs (**Figure S1**). Red bar indicated the number of voxels on VOIs in the group with tumors in the right hemisphere (RH Group); green bar indicated the number of voxels on VOIs in the group with tumors located in the left temporal area (LT Group); and blue bar indicated the number of voxels on VOIs of the patients with tumors in the left frontal area (LF Group). BOLD activation decreased progressively on VOI 1 and VOI 4 from RH Group, LT Group to LF Group. In contrast, activation increased progressively on VOI 3 in the right hemisphere. BOLD activation on Wernicke’s area (VOI 2) was mostly diminished in LT Group, but was increased in the superior parietal gyrus on both hemispheres (VOI 5 and VOI 6).

***:** *P* < 0.05 when comparing RH group (tumors on the right hemisphere).
